# Vapor Pressure of Water at Its Triple Point

**DOI:** 10.6028/jres.080A.054

**Published:** 1976-06-01

**Authors:** L. A. Guildner, D. P. Johnson, F. E. Jones

**Affiliations:** Institute for Basic Standards, National Bureau of Standards, Washington, D.C. 20234

**Keywords:** Precision mercury manometer, triple point of water, vapor pressure of water

## Abstract

The vapor pressure of water at its triple point was measured with exceptionally high accuracy by realizing it with a special apparatus and measuring the pressure with the NBS precision mercury manometer. The vapor pressure apparatus had a system for circulating the liquid water. Actual triple point conditions were established with a thin sheet of freshly distilled liquid flowing down over an exposed mantle of ice frozen on a vertical well. This technique reduced non-volatile contaminants and the vapor was repeatedly pumped to remove accumulated volatile contaminants. A diaphragm pressure transducer was used to separate the water vapor from the helium used to transmit the pressure to the manometer. The value found for the vapor pressure of water at its triple point was 611.657 Pa with an uncertainty of ± 0.010 Pa from random errors, computed at 99 percent confidence limits. The systematic errors are estimated to be insignificant relative to the random errors.

## 1. Introduction

Water is the stuff of life—the working medium for power generation—the great highway of commerce — the determining factor in the weather. It has such importance to mankind that probably no other single compound has received more attention from the scientific world. Its properties have been measured and remeasured; thermodynamic tables have been constructed and repeatedly revised, and further work of this type is even now commanding worldwide attention. In part, this kind of activity continues because water is not an easy substance to deal with. It is almost a universal solvent, often reacts with the materials of an experiment, and is susceptible to significant changes in its isotopic composition. Consequently, high accuracy in the measurement of many of the properties of water is hard to achieve, and more effort is needed to extend or improve our knowledge of them.

It is our purpose to measure with the highest possible accuracy the vapor pressure of water for – 5 °C < *t*_68_ < 100 °C. We chose to concentrate initially on the vapor pressure of the triple point, which was known with poor accuracy. Although a “triple point” is a precise designation for a phase diagram, it is not likely to be an actual point in its physical realization. Thermodynamically, a triple point is a system containing matter in three phases with each component in equilibrium throughout the system, i.e., the chemical potential of each component is the same in every phase. If all three phases should happen to be in contact simultaneously (which is not a requirement of the definition), the contact will be along a line producing a closed curve, reducing to a point only in the limit. Furthermore, there are several triple points of water. We shall use the unqualified expression, however, to denote the equality of the chemical potential of pure water in a system containing the three phases: ice I, liquid and vapor. This is the only triple point of water involving the 3 states of matter and the one of most importance. Not only is it the terminus of the ice I-vapor pressure curve and the beginning of the equilibrium liquid-vapor pressure curve,^1^ but also its temperature, which can be expected to be invariant by the phase rule, has been assigned a value that, in principle, establishes all thermodynamic temperatures. This unique situation makes a highly accurate value of its vapor pressure, which is also invariant, especially interesting, because it can provide the foundation for exceptionally accurate thermodynamic calculations for water. This is true for this temperature region in particular because thermodynamic temperatures are inherently known with their highest accuracy in the region of the defining value.

Most values reported for the vapor pressure of the triple point have been derived by interpolation or extrapolation of measurements of the two-phase equilibria between the liquid and vapor as a function of temperature. The most significant of these are the values of Thiesen and Scheel, 610.90 Pa [1],^2^ Scheel and Heuse, 610.90 Pa [2] and 610.55 Pa [3] and of Douslin, 612.1 Pa [4]. The average of the earlier results fitted along with data over a large temperature range is perhaps best represented, in the sense of a consensus, by the accepted value of the triple point in present day steam tables, viz., 611.2 Pa.

For equal accuracy of pressure measurements, those workers who actually realized the triple point probably attained greater accuracy in the measured vapor pressure. There are only two papers reporting such measurements:

1. The values of Prytz, published in 1931 [5], had an average of 4.5867 mm of Hg pressure, or 611.51 Pa.^3^ He realized the triple point statically, i.e., an incomplete layer of ice was formed on the surface of a quiescent pool of water. The pressure was measured by a mercury manometer using interference techniques to locate the menisci with 0.5 *μ*m Hg (0.065 Pa) imprecision, but the values of the vapor pressure varied by as much as 1.7 *μ*m Hg (0.22 Pa). Prytz was of the opinion that the average value was about 2 *μ*m Hg (0.26 Pa) too high.
2. L. Besley and G. Bottomley published two values, 611.29 Pa obtained from direct measurement and 611.11 Pa derived by interpolation [6]. A special mercury manometer was used for the pressure measurements, with the height of the mercury column determined cathetometrically. The vapor pressure cell was connected directly with the manometer, as was also the case in the measurements by Prytz. Considerable effort was made to eliminate dissolved air in the filling of the cell. It was operated statically, being carefully thermostated to realize the 3-phase equilibrium at the surface of the liquid. The total uncertainty of either value was not given. The value derived from direct measurements was based on 148 determinations which varied between 4.579 and 4.590 Torr, and one might thereby regard the variation of ±5.5 m Torr as equivalent to the imprecision at the 99.4 percent confidence level. The residual standard deviation for the polynomial, from which the interpolated value (4.5837 Torr) was derived, was 1.7 m Torr. The two imprecisions are not inconsistent, insofar as three residual standard deviations would approximate the imprecision of the interpolated value at the 99+ percent confidence level, except that in addition the uncertainty would be greater because the triple point was near the lower extreme of the range of the fit.

Our own method also realized the triple point, but in a dynamic system. Liquid water is distilled onto a mantle of ice and falls into a well to be pumped back to the still. This method helps to continuously reduce the concentration of dissolved gases which come out of solution in the still and in the pressure cell and are pumped off periodically. Since the evaporation from the still is quiescent, nonvolatile impurities are retained in the boiler. To maintain the purity of the water vapor in the triple point cell, and of the helium in the manometer line, the vapor pressure system is separated from the manometer line by a diaphragm pressure transducer.

## 2. Equipment

Improved accuracy of the measurements of the vapor pressure of water at the triple point can only be attained by:

1. Making more accurate pressure measurements.
2. Realizing the triple point better.
3. Improving the purity of the water, especially with regard to gases in solution.

**Figure 1:**
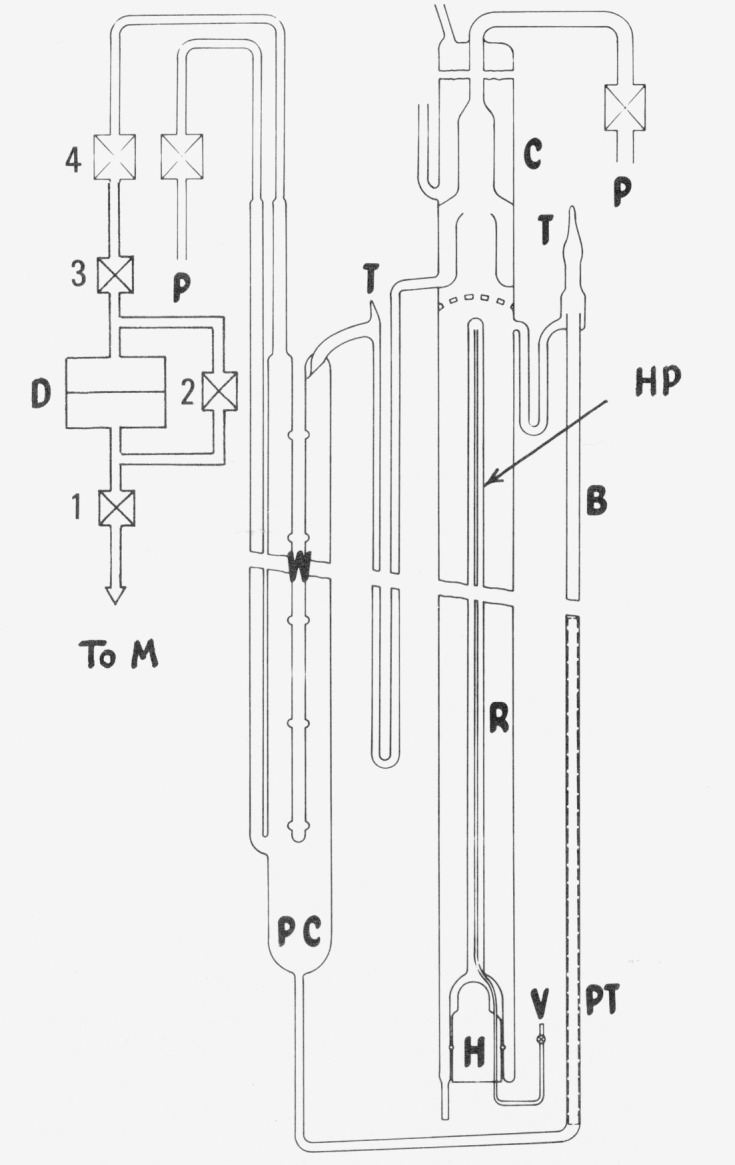
Schematic of equipment.

The measurements were undertaken in the NBS Gas Thermometry Laboratory because the first problem was essentially solved by using the NBS precision mercury manometer to measure the pressure. The second and third problems were dealt with by the construction of an elaboration of the triple point cell, and by the use of a metal diaphragm pressure transducer. The arrangement of the equipment is shown schematically in figure 1, where M is the manometer, D is the diaphragm, and the remainder is the valving and the triple point cell. We shall discuss the equipment in terms of these three main elements.

### 2.1. The Manometer

The principles and many of the details of the NBS precision mercury manometer are given in an earlier paper [7]. Essentially it is a W-tube manometer, with the locations of the crowns of very large menisci precisely reproduced by the use of capacitance measurements, and with the height of the mercury column accurately measured by end length standards (gage blocks). The instrumentation of the manometer was designed so that a null signal on a capacitance bridge was observed when the gas pressure in the lower cells balanced the pressure from the mercury column plus the vapor pressure of the mercury in the upper cell. The maintenance of the pressure was made automatic by using the output of the bridge to control a heater in a large ballast (a “thermal injector”) so as to restore the pressure.

**Figure 2:**
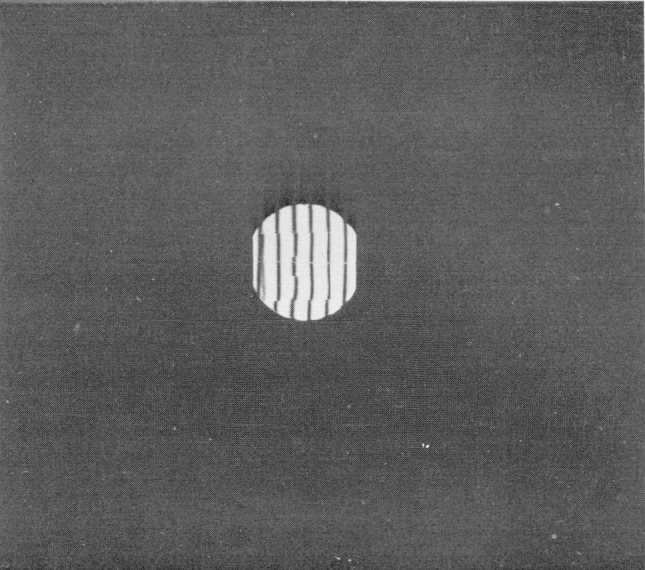
Interference pattern of gage block One fringe equals about 0.25 *μ*n.

The pressure to be measured is between 611 and 612 Pa, corresponding to a height of the mercury column close to 4.6 mm. We bought a chromium carbide gage block of that length in the highest quality offered. It was carefully calibrated by J. S. Beers, Deputy Chief for Length in the Dimensional Technology Section. In figure 2 the interference pattern of this block indicates very good flatness and parallelism, with one corner slightly low relative to the gaging point. These properties make meaningful the high precision of the calibration measurement, which had an estimated standard deviation of the mean of 2.6 × 10^−6^ mm. This includes the variability due to wringing.

**Figure 3:**
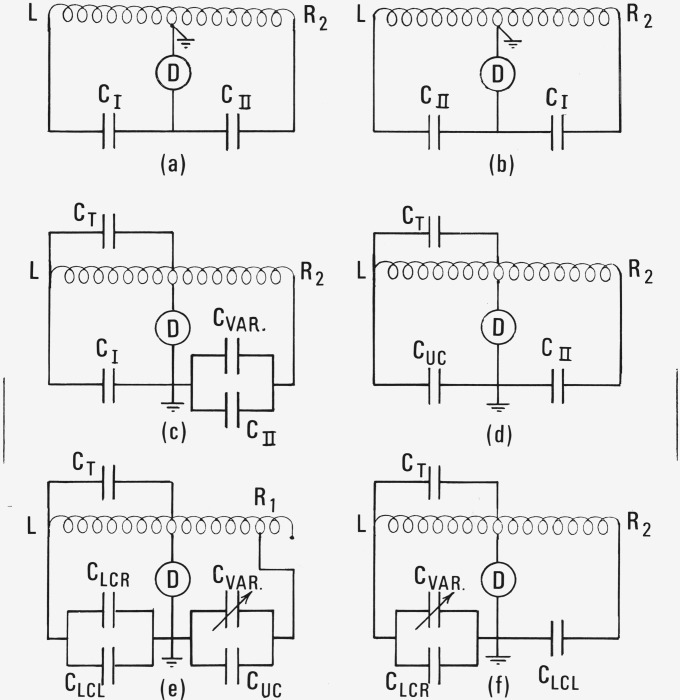
Configurations of circuits used to measure the pressure.

Because it is impracticable to obtain a gage block of exactly the correct length (particularly in advance of knowing its value) some modification in our usual method of measuring pressure was necessary to provide continuity over the interval between gage block lengths. There is too much uncertainty introduced by measuring an appreciable pressure difference directly by the diaphragm gage. However, the characteristics of the manometer cells are well known, so that an accurately known change of capacitance can be accurately related to a change of meniscus height. The bridge is balanced at “zero level” by adjustment of *C*_Var_, a 3-lead General Radio variable capacitor, Type 1422–CE,^4^ used in the range 0.005 to 0.11 pF, as shown in figure 3e. This capacitor has an imprecision of 0.0001 pF and was calibrated at the values used with an uncertainty of the reference estimated at ±0.00003 pF. The uncertainties in the effective areas of the capacitance plates (±0.03% at 99% confidence) limit the attainable accuracy in calculating the change of meniscus height from a change of *C*_Var_. However, so long as the height of the mercury column differs from the gage block length by no more than 17 *μ*m, the contribution to the error in the measured vapor pressure from this cause will not exceed 1 ppm.

### 2.2. Modified Triple Point Cell

The vapor pressure apparatus, shown in figure 1, provided for circulation of the water. It was pumped from the pressure cell, PC, by a bubble pump, B into a reservoir, R. The reservoir was part of a still with a heater, H, to evaporate water into a condenser, C. The condenser water reentered PC at the top through a trap. The cell was 38 cm deep, with a volume of about 500 cm^3^. A thermometer well, W, 32 cm deep, was a close fit on the 7 mm diameter case of a platinum resistance thermometer. Seven bulges, 20 mm across, supported the ice mantle when the water was pumped out of the cell. The bubble pump was a vertical tube of 5 mm bore with a piece of perforated PTFE (polytetrafluoroethylene), *PT*, inserted in the lower half. A 50-watt strip heater was taped to the tube and thermally insulated from the bath. The reservoir was 50 mm in diameter and had a volume of about 700 cm^3^. It had a reentrant tube HP, which extended to the top of the boiler. The tube contained C_2_Cl_3_F_3_ and functioned as a heat pipe, transferring heat from the heater, H, to the water surface. It was filled through the valve, V. There were three tubulations: one from the top of the cell to valve 4 was for the pressure measurements, and two others, *P*, one from the side of the cell and one from the condenser, were for pumping from the gas phase. The tubulations were sealed by PTFE gaskets to stainless steel high-vacuum valves at the top of the assembly.

### 2.3. Pressure Transducer

A special commercial pressure gage with a metal diaphragm, the position of which was sensed on one side by the capacitance between it and an electrode, was used as a null device to detect equality of pressure between the water vapor on the one side and the helium gas transmitting the measured pressure on the other side. The null reading could be determined before and after measurements by opening the bypass, valve 2 in figure 1, and adequately evacuating both sides. Great care had to be taken in the use of the instrument to achieve the needed stability, both electronic and physical.

## 3. Filling of Vapor Pressure Apparatus

The vapor pressure apparatus was cleaned and filled by the same techniques used in the preparation of conventional triple-point cells. The glass was cleaned with chromic acid, rinsed, lightly etched by hydrofluoric acid, and leached by steaming. Two vapor pressure cells were filled in the course of a production run of triple point of water temperature standards, with water that was purified first by treatment with an ion exchanger and then quadruply distilled, with chemical treatment for removal of organic material between each distillation.

Samples of the purified water were taken for isotopic analysis at the time of filling and also later during operation. Up to the present, there is no measurement of the vapor pressure of the triple point of naturally occurring water for which the maximum reported variation of isotopic composition would make a significant difference. However, the high accuracy of the present measurements being reported in this paper require such a specification to avoid added uncertainty.

## 4. Procedure

The operation of the equipment consisted of preparing the manometer and the vapor pressure apparatus for measurements, determining the null of the evacuated diaphragm gage with the bypass valve open, and then, after closing the bypass valve, back filling the diaphragm with helium on the side of the capacitor electrode and with water vapor on the other. The vapor pressure apparatus was then vacuum pumped for 30 s in each of three successive 2 min periods before any actual measurements were made. We shall expand on these preliminaries and then describe the measurements themselves.

The manometer is “zeroed” with a fixed mercury-to-capacitance-plate separation in the upper cell and for the corresponding separations in the lower cells when all three mercury menisci are on the same level. Under these conditions, the “zero level” capacitance necessary to balance the capacitance bridge (see fig. 3e) is established. The measurement of a vapor pressure consists of generating and measuring in the manometer the equivalent pressure of helium, as evidenced by a zero pressure difference at the diaphragm pressure transducer. To do this, a gage block of appropriate height is inserted between the upper cell and its pedestal, and the pressure of helium in the lower cells and the volume of mercury in the manometer are adjusted until all mercury menisci are at a level relative to the capacitance plates to produce the same capacitances as for zero level. In these measurements, for the first time, we have modified this procedure in order to interpolate between the gage block values. We adjusted the manometer pressure until the diaphragm was balanced, and then we balanced the manometer capacitance bridge by adjusting *C*_Var_.

The vapor pressure apparatus when not in use is operated in a “Standby” mode, in which the water is continuously circulated by pumping it out of the triple point cell into the still and distilling it back into the triple point cell. Occasionally, the vapor is vacuum pumped, the purpose being to remove gases released from solution, The operating parameters, and even some details of design, of this apparatus were determined by long experimentation. The heat pipe in the condenser reservoir was necessary to avoid overheating the water near the bottom of the reservoir. With the apparatus in a 0 °C thermostat and a heater power of 25 watts, the water temperature in the reservoir was about 25 °C and nearly uniform up to 1 cm below the surface. Distillation occurred at the rate of about 0.01 g/s. The bubble pump, operated with a heater input of 15 W, initially transferred a mixture of water and vapor from the full triple point cell to the reservoir at a rate of about 10 g/min, but the rate dropped as the water level dropped. When the cell was nearly empty, the pump acted as a still with a transfer rate of a few g/h.

To prepare for measurements, the apparatus was placed in a bath, thermostated by circulating liquid close to the triple point temperature and constant within 0.2 mK. The vapor pressure cell was filled by distillation from the condenser (operated at 20 W this required about 24 h). Bath fluid was circulated through the condenser, and was pumped over the top of the cell and also onto the stem of a platinum resistance thermometer inserted into the well of the vapor pressure cell. (When water was being pumped out of the cell, a zone at the top of the reservoir was cooled by pumping bath fluid over it also).

The apparatus was removed from the bath and a mantle of ice was frozen around the thermometer well using powdered solid CO_2_ as the coolant. The completed mantle was from 5 mm to 10 mm thick.

The apparatus was then returned to the bath, which was regulated at a temperature not more than 1 mK below the triple point temperature and the bubble pump was started. By the next morning, the cell was nearly empty whereupon the reservoir heater was turned on. It distilled water into the pressure cell at about 0.01 g/s (with the bubble pump left on, measurements could be conducted for several hours before enough water accumulated in the cell to touch the ice mantle).

In the meantime, both sides of the diaphragm gage had been evacuated overnight with an ion pump. A “zero” reading was obtained with the gage isolated and the bypass valve open. Then the bypass valve was closed, the electrode side of the diaphragm cell was filled with helium, and the other with water vapor (with the differential pressure across the gage kept within ±1 mm Hg). Finally, adjustments of the manometer variable capacitor were made in order to balance its bridge when the manometer pressure was such that the reading on the diaphragm gage was very near the “zero” value.

Next, to sweep out gaseous impurities, the water vapor was pumped from the pressure cell, at a rate of approximately 100 cm^3^/s (0.6 cm^3^ S.T.P./s). When the apparatus was in regular use, an initial set of three pumpings, each pumping lasting 30 s over a period of 6 min was sufficient to insure no effect after recovery to a steady state would be observed with further pumping.

Chart records were made of the diaphragm gage output for its zero, and immediately following the last pumpout, readings of the output of the diaphragm gage were recorded. Because the stirring of the thermostat had to be stopped to avoid shaking the diaphragm, the recordings were made for 20 s periods every two minutes. After each set of 3 recordings, the water vapor was again pumped for 30 s. After 4 such sets, the apparatus was closed off, the diaphragm bypass valve opened, and the diaphragm pumped out for 20 min when a new “zero” was recorded. During each measurement, the temperature of the diaphragm was sensed by reading the e.m.f. of a copper-constantan thermocouple referred to ice.

## 5. Measurements

Besides general experimentation to establish effective operating techniques, there were three periods of several weeks during which actual pressure determinations were made. The first period was purely instructive and resulted in vigorous efforts to improve various aspects of the equipment, in particular the diaphragm gage. The results of the second period reflected substantial improvement in the accuracy of the measurements, and were reported at the 1974 meeting of the International Association for the Properties of Steam in Giens, France [8]. The results of the third period are decidedly more accurate than the second, more because of further improvement in the diaphragm gage than anything else. However, numerous possible effects were investigated in addition, and these results will be described here before presenting the final sets of measurements.

Three standard calibrated platinum resistance thermometers were used to measure temperature, one in the well of the vapor pressure cell, the second to measure the temperature of the thermostat bath close to the vapor pressure cell, and the third as a regulator sensor. They were calibrated in a triple point of water cell each day; it is believed the values of the measured temberatures were uncertain by no more than 30 *μ*K. The temperature registered by the thermometer in the well of the vapor pressure cell could be used as an indication of the state of the mantle. When the ice mantle was dry or nearly so, pumping resulted in a rapid reduction in the pressure, by about 100 Pa, and in the temperature, by about 0.1 K. When an adequate flow of water was maintained over the ice during pumpout, the drop in pressure was no more than 0.5 Pa and in temperature no more than 0.1 mK.

It has also been observed during a run earlier than any being reported that when air in an amount > 100 mm Hg in pressure had entered the cell, the temperature at the mantle was depressed by several tenths of a mK and did not recover fully before a new mantle was frozen.

The effects on the observed vapor pressure resulting from variation of the bath temperature from the temperature of the triple point were studied. For temperatures in the range from 1 mK below the temperature of the triple point to 200 mK above it, scarcely any variation of the vapor pressure was observed. This indicated that the temperature surrounding the apparatus was not very critical, and furthermore, there was probably no undesirable effect from the warmer parts of the tubulations which extended out of the bath. In the final measurements, the bath was thermostated at, or not more than 1 mK above, the temperature of the triple point of water.

The diaphragm unit itself was not thermostated, but was thermally lagged with a thick layer of insulation. Its temperature was measured at frequent intervals, and in its final physical and electronic configuration, a consistent relationship between the copper-constantan e.m.f.’s and the zero readings existed. A straight line was fitted to the data by the method of least squares with the result that the zero can be calculated from the e.m.f. by the following equation: *y* = 893.627 – 0.748503 V where *y* is the chart reading for the diaphragm zero and V is the thermocouple e.m.f. in *μ*V. The residual standard deviation was 1 chart division, and the standard deviation of a predicted point was about 0.5 chart division. This equation was used to calculate the chart zero pertinent to any given chart pressure reading, from thermocouple e.m.f.’s observed at the same time.

No effect dependent on refreezing the mantle was observed, and in fact, so long as crystals of ice remained, it appeared that the pressure was established, except for the change in the “pressure head”, i.e., the pressure due to the weight of the column of vapor above the effective location of the line at which triplepoint conditions exist.^5^

The values given in this paper were measured on four successive days and consist of the following five “sets”:

1. Set 1: Ten values of diaphragm gage readings in 3 groups, with zeroes before and after the readings.
2. Set 2: Nine values of diaphragm gage readings in 3 groups, with zeroes before and after the readings.
3. Set 3: Eight values of diaphragm gage readings in 3 groups, with zeroes before and after the readings.
4. Set 4: Twelve values of diaphragm gage readings in 4 groups, with zeroes before and after the readings.
5. Set 5: Twenty-five values of diaphragm gage readings in 8 groups, with zeroes before and after each 4 groups.

A group comprises the values measured between pumpouts. After the initial three 30 s pumpouts, the apparatus was pumped about every 10 min. At the conclusion of all the measurements, the value of the capacitance to balance the capacitance bridge for the manometer at zero level was redetermined.

## 6. Equations and Calculations

Because of the effect of the pressure head, there is only a surface, a horizontal plane except for temperature perturbations, at which the triple point pressure can exist. The pressure of the water vapor at the line formed by the intersection of the surface with the mantle is given by the following:

$$P_{tp}=P_{\text{Man}}+\delta P_{\text{Wring}}+\delta P_{\text{CVar}}+\delta P_{\text{Thermp}}+\delta P_{\text{Dia}}+\delta P_{\text{Vap}\;\text{Hd}}.$$

*P*_Man_ is given by eq (27) of ref. [7], with temperatures for zero level equal to 20 °C and for helium filling gas. The vapor pressure of mercury and its temperature derivative have been reevaluated at 20 °C as 0.171 Pa and 0.0147 Pa K^−1^, respectively, and the density at zero pressure is evaluated for *t*_68_ = 20 °C (rather than *t*_48_), as 13,545.82 Kg/m^3^ [9]. Symbolically,

$$\begin{array}{l} P_{\text{Man}}=-\alpha \rho g(h_{UC}\delta t_{UC}-{\bar{h}}_{\text{LC}}\bar{\delta }t_{\text{LC}})+{\left(\frac{dP}{dT}\right)}_{\text{Hg}\;\text{Vap}}(\delta t_{\text{UC}}-20)\\ \;+\rho gh_{2}(1-\alpha [\delta t_{\text{Hg}}])+P_{20}(\text{Hg})\\ \;+\rho gh_{2}(2.27\times 10^{-6}h_{2}-2.5\times 10^{-7}-4.07\times 10^{-3}Mh_{0}), \end{array}$$

where *α* is $\frac{1}{V}{\left(\frac{\partial V}{\partial t}\right)}_{P}$ for mercury, *ρ* is the density of mercury under zero pressure at 20 °C, *g* is the acceleration due to gravity, the *δt*’s are differences of temperature from 20 °C of the upper cell (UC), the average of the lower cells (LC), and the mercury arm (Hg). The height of mercury in all the cells is substantially the same and is symbolized by *h*_UC_ for the upper cell or by ${\bar{h}}_{\text{LC}}$ as the average for the lower cells. The height of the mercury column is *h*_2_, and the pressure head is produced by the height of the gas column between the menisci of the lower cells and the center of the diaphragm (which was mounted vertically), designated *h*_0_. The pressure head depends upon the molecular weight M. The numbers are consistent for values of the symbolic quantities in S.I.

“Manometer readings,” comprising the observations of the resistance of a capsule platinum resistance, thermometer located in a thermocouple reference block and the e.m.f.’s of all 4 sets of differential thermocouples for the upper cell, the mercury arm, the left lower cell and the right lower cell, were made once or twice for each set of vapor pressure measurements. The temperatures of the vertical components of the mercury lines were calculated from the thermocouple e.m.f.’s as a difference from the temperature of the reference block. There were twelve copper-constantan thermocouples in series fastened to the upper cell, twenty-five to the mercury arm, ten to the left lower cell and twelve to the right lower cell. The observed e.m.f.’s were converted to temperature differences by a “handbook” sensitivity of 40.5 *μ*V °C^−1^, per junction at 20 °C, viz., 486, 1013, 405 and 486 *μV* °C^−1^, respectively. The platinum resistance thermometer qualified as a standard instrument for the IPTS, with a reading in the triple point cell of *R*_PRT_/*R*_Std_ = 0.25557275, and calibrated values of

$$A=3.9855387\times 10^{-3}{\;}^{\circ }C^{-1}$$

and

$$B=-5.8755669\times 10^{-7}{\;}^{\circ }C^{-2}.$$

The values of temperatures were calculated by the program BRIDGE, given in appendix I. The following results (table 1) were obtained when the experimental data were calculated by the program PRSURE, given in appendix II.

The manometer zero was established from 10 determinations made over a period of 10 days. The values were adjusted to that which would have been observed if all parts of the manometer were at a uniform temperature of 20 °C. The equation is derived from eq (27) of ref. 7, and as programmed in Basic is given in appendix III. The results in Pa were converted to nm of Hg and in turn the value of the variable capacitor was adjusted on the basis that a change of 1 nm is equivalent to 1 × 10^−5^ pF. The values measured and the calculated results were as follows (see table 2):

*δP*_Wring_ is the net correction for imperfectly joined gaging surfaces between the wringing boss on the bottom of each cell and the pedestal or the gage block. The leak rate through the gap formed when two gaging surfaces meet on one edge with a dihedral angle *ϕ* (in *μ* rad) was studied [10] and is given for the particular blocks and volume of vacuum system as

$$dp/dt=0.42\phi^{2}+0.22\phi^{3}\mu m\;\text{Hg}/\min.$$

The vacuum leak rates were measured, both during the determination of zero level and several times during the course of the vapor pressure measurements. The gaging surfaces are square, of a width *W* = 2.41 cm on a side. The change in height between the measurement of zero level and the pressure measurement was then

$$\delta h=[\phi (\text{UC})-\phi_{0}(\text{UC})-\{\phi (\text{LCL})+\phi (\text{LCR})-\phi_{0}(\text{LCL})-\phi_{0}(\text{LCR})\}/2]\times W/2$$

and, with δ*h* in nm, the pressure effect in pascals is

$$\delta P_{\text{Wring}}=\delta h\times 1.32763\times 10^{-4}\text{Pa}.$$

The leak rates were measured for each cell separately. It was calculated that the volume of each cell plus thermocouple gage is related to the total volume from which the original equation was derived as

$$\begin{array}{l} V_{\text{LCL}}/V_{\text{Tot}}=0.579\\ V_{\text{LCR}}/V_{\text{Tot}}=0.620\\ V_{\text{UC}}/V_{\text{Tot}}=0.523. \end{array}$$

The rates were remeasured after every adjustment of any cell position. The net pressure effect is given in the following:

δ*P*_CVar_ is derived from the change of mercury level requiring a change of the variable capacitor to maintain the manometer bridge balance. The change of level can be accurately determined because of the coaxial switching arrangement that is part of the manometer instrumentation. The configurations used are shown in figure 3. The manometer transformer has two taps on the right-hand side, and one on the left. The tap *R*_1_ has 3/4 the voltage of *L*, and *R*_2_ has the same voltage as *L.* The variable capacitor may be switched to any tap.

There is also a switch which changes ground from the center tap to the other side of the detector. There are 3 standard capacitors, labeled *C*_I_, *C*_II_, and *C*_III_. The values of *C*_I_ and *C*_II_ are 8.2046 ± 0.0008 pF, and are equal within 1 ppm. The value of *C*_III_ is 3.0503 pF, so that it can be used to measure the lower cell capacitances. The capacitances of the various parts of the detection system and the manometer were found from the following sets of measurements at zero level:

The mercury cell capacitors comprised guarded circular plates facing a grounded surface that was essentially infinite. The capacitance can then be expressed as *C=* 1.11267 A/(4 *π* S) pF, where A is the effective area of the plate (in cm^2^), and S is the separation between the plate and the mercury (in cm). The dimensions of the cells are accurately known; the following separations at zero level can then be calculated (table 5):

In operation, the mercury level is maintained so that *C*_UC_ = 8.16092 pF. The value of the variable capacitor to balance the bridge at zero level, the “manometer zero,” corrected for differences of mercury temperature from 20 °C, was 0.02435 pF. The vapor pressure was measured with the variable capacitor in the L arm, so that *C*_T_ + *C*_LC_ + *C*_Var_ = 3/4 *C*_UC_ and the change of capacitance of the lower cells from the balance at zero level is 0.75 × 0.02435 pF plus the reading of *C*_Var_ for the Set. This change of capacitance corresponds to a displacement, *d*, in the separation of the mercury surfaces from the plates in the lower cells, which is

$$\Delta \sum C_{\text{LR}}=\frac{1.11267}{4\pi }\left[A_{\text{LCL}}\left(\frac{1}{S_{0}(\text{LCL})+d}-\frac{1}{S_{0}(\text{LCL})}\right)+A_{\text{LCR}}\left(\frac{1}{S_{0}(\text{LCR})+d}-\frac{1}{S_{0}(\text{LCR})}\right)\right].$$

The pressure in pascals is related to the displacement in *μ*m as

$$P_{C\text{Var}}=0.132763\times d\;\text{Pa}.$$

The values obtained are given in table 6.

δ*P*_Thermp_, the effect of thermomolecular pressure, is expected to be significant only in the pressure transmitting tube of the manometer. This tube, which is 0.1512 cm in radius, ran from the manometer vault, at a temperature very close to 20 °C, to the valves that were physically adjacent to, and assumed to be at the temperature of, the diaphragm unit.

The value of the effect was derived from the equation of Weber, Keesom and Schmidt [11] (the program in Basic for their equation is given in appendix IV). We measured the thermomolecular pressure difference of helium in a tube of 0.0412 cm radius and found the values to be less than the values from Weber's equation by a factor of 0.76. The functional relationship in the Weber equation for the radius of the tube, i.e., that *p*Δ*p* ∝ 1/r^2^, is assumed to be correct. The difference between theory and experiment is thought to occur because of the inadequacy of other assumptions. Therefore, the same factor of 0.76 was used in the calculation because it should still be applicable for the larger tube. (See table 7.)

δ*P*_DIA_, the pressure difference at the diaphragm, was derived from sets of charts readings. Following the stated operating procedures, we recorded the diaphragm zero and the copper-constantan thermocouple reading, then pumped three times, after which three sets of measurements, each set consisting of a thermocouple reading and a diaphragm reading for the pressure difference, were recorded. The diaphragm output was recorded for 20 s every 2 min. The thermocouple was read again while the water vapor was repumped from the vapor pressure cell. Three more thermocouple and pressure readings were taken for 20 s at 2 min intervals, and then the diaphragm was evacuated for 20 min and a diaphragm zero recorded. It was experimentally demonstrated that this period of evacuation of the diaphragm was long enough to assure reliable zero values.

Within the imprecision of the instrument, the diaphragm readings were correct on the 10 *μ*m Hg scale, so that the chart measurements could be calibrated by the diaphragm readings. In order that the uncertainties would be small, the manometer was adjusted so that the difference of the diaphragm from zero was small. The net deflections in chart divisions in Set 1 were – 2, – 2.2, – 1.7, – 6.6, – 4.7, – 1.7, – 5.5, – 3.7, – 4.7, and – 5.4, for an average of – 3.82 (*S =* 1.65). For set 2, they were – 0.2, 0.8, – 0.8, 0.8, 2.8, 2.5, 2.5, 2.5 and 1.5, for an average of 1.38 (*S* = 1.31). For set 3, they were 1.1, 0.1, 0.9, 1.5, 0.5, – 1.5, – 1.2 and – 0.8, for an average of – 0.12 (*S=* 1.04). For set 4 they were 1.1, 2.2, – 0.1, – 2.1, 1.2, – 1.7, 2.8, 4.3, 0.3, 1.3, – 0.1, 0.6, for an average of 0.82 (*S* = 1.79). For set 5 they were 2.5, 1, 3.4, 0, 2.5, 1.5, – 0.5, 1.5, 1.5, – 0.5, 2.2, 1.2, 3.1, 2.1, 0.2, – 1.8, – 1.3, 0.2, 1.1, 1.1, 0.6, 2.6, – 1.9, 0.2 and 1.6, for an average of 0.89 (*S=* 1.39).

The chart calibration gave a sensitivity of 0.00536 Pa/div so that we have for the five sets (with S denoting the estimate of the standard deviation for a single measurement and *S_m_* the estimate of the standard deviation of the mean) (see table 8):

δ*P*_VapHd_, the pressure head produced by the column of water vapor, is calculated from the estimated vertical distance, *h*, from the midpoint of the diaphragm to the line at the mantle where the triple point is realized. We believe this line to be near the top of the mantle, but its position is not entirely stable, both because the flow of water may vary and because the mantle melts and recedes from the top. The pressure is calculated as

$$\delta P_{\text{VapHd}}=\frac{P_{\text{Man}}\;g\;M}{RK}\left(\frac{h_{1}}{T_{1}}+\frac{h_{2}}{T_{2}}\right),$$

where *g* is the acceleration due to gravity, *M* is the molecular weight of the vapor, *R* is the molar gas constant, *K* is the conversion factor between units of pressure, *h*_1_ is 0.244 m running between the diaphragm at 25 °C and the bath at 0°C, and *h_2_* is the distance, 0.07 m, in the bath at 0 °C. Then

$$\delta P_{\text{VapHd}}=23.31\times 10^{-6}P_{\text{Man}}=0.01424\;\text{Pa}.$$

The average temperature, *T*_1_, was weighted on the high side. The value is bounded by the extremes, of course, and within those limits any reasonable choice would hardly make any significant difference in the final pressure.

## 7. Results

The results are summarized by totalling the elements of pressure for each set (all values in pascals):

## 8. Isotopic Composition

The determination of the isotopic content of samples from the vapor pressure cell relative to SMOW (standard mean ocean water) depended upon two steps. First, the isotopic content of cell water was determined relative to NBS-1 standard water [12], the composition of which had, as a second step, been related to SMOW by the work of Craig [13]. The *D/H* absolute abundance ratio of SMOW has been determined very precisely by Hagemann, Nief and Roth as 155.5 ppm [15]. The value for the absolute abundance of O^18^ in SMOW is based on Craig's measurements [13, 14] and as variously interpreted may be taken to be O^18^/O^16^ = 1993.4 × 10^−6^ to 1995 × 10^−6^, of which the latter is used here. The compositions of seven samples of the water used in the vapor pressure cell, both relative to SMOW and absolute, are as follows:

All measurements were made with the second cell. The difference between the vapor pressure of the cell water and SMOW because of the reduced compositions of D and O^18^ can be calculated from measurements of Majoub [16], who reported that the ratio of $P_{\text{HDO}}/P_{H_{2}O}$ at 0°C is 0.8994, and of various authors [16, 17, 18], who reported ratios of $P_{H_{2}O^{18}}/P_{H_{2}O^{16}}$ at 0 °C that average about 0.9885. The difference of pressure may then be derived from Raoult's Law, where the pressure of the liquid of the sample at 0.01 °C is 1 ppm more than the corresponding metastable SMOW. The sample triple point temperature is about 40 *μ*K lower than the triple point of SMOW [19]. Given that *dP/dt* is about 44.4 Pa/K for water at 0.01 °C, the effect of the difference in temperature of the triple points is about 3 ppm. Thus the vapor pressure at the triple point of the sample, which corresponds closely to the analyses of typical “continental water,” can be calculated to be about 2 ppm, or 1.2 m Pa, less than the vapor pressure at the triple point of SMOW.

## 9. Discussion

It is, of course, crucial to the reliability of any data that procedures be established to eliminate or satisfactorily minimize possible errors before any final measurements are made. We believe that the probable difficulties requiring special study were (1) the possible variation of the vapor pressure because of a slow return to equilibrium following a pumpout, or with variation of ambient temperature, (2) the presence of contaminating gas in the system, and (3) the possible errors in determining the diaphragm zero. Each of these problems received special study.

As discussed in section 5, the pressure and temperature were only slightly perturbed by pumpout so long as all three phases were maintained about the mantle. Similarly, in the presence of the mantle, there was at most only a slight change in vapor pressure when the ambient temperature varied from 1 mK below to 0.2 K above the triple point. Thus the actual realization of the triple point must have contributed great stability to the vapor pressure; the broad expanses of exposed liquid and solid phases could be expected to fulfill the physical necessities for facilitating such an equilibrium.

The procedure of pumping for three 30 s intervals prior to measurements, and subsequently for 30 s every 10 min was adopted because the measured pressure, with lesser amount or frequency of pumping, varied in a way to indicate the accumulation of measurable amounts of contaminating gases. The three recorded diaphragm pressure readings made in each interval between pumping showed no evidence of preferential drift that might indicate the rate of pressure buildup to be significant in that span of time.

An accurate measurement of the diaphragm zero depended upon the evacuation of enough of the sorbed gases (particularly water vapor) that the remainder would not exert a significant pressure difference between the two sides of the diaphragm, with the by-pass valve open. It was observed experimentally that a 20 min evacuation following pressure measurement appeared to be sufficient. These measurements together with the thermocouple e.m.f.’s observed at the same time correlated with those values of zeroes after long pumping and their concomitant thermocouple e.m.f.'s. This fact offered substantial confirmation both that 20 min was an adequate pumpout and that the diaphragm zero could be accurately associated with the thermocouple readings. However, measurements made when the room temperature varied rapidly were not in agreement with the rest of the results, and were not used. The assumption was that the thermocouple, being on the outside of the diaphragm, could not have represented the appropriate temperature when there were substantial gradients.

The reader may note that the corrections for *C*_Var_ appear to be inconsistent. This is because a different reference line for reading was used at different times.

## 10. Estimation of the Total Uncertainty

The stated total uncertainty consists of the limits, at 99 percent confidence level for the random errors; and the systematic errors, estimated conservatively enough to warrant about the same confidence level.

The estimates of the values of the errors are expressed initially as one standard deviation. It is useful to treat them in two groups: (A) Those sources of error which contribute to the imprecision calculated from table 9, and (B) those additional sources of error which have a fixed effect on the directly determined value.

The term "systematic error" can be understood, and is used by some authors, to denote any error that introduces a constant bias into the observed results. For example, this is true of each of the items of Part B. However, in all but the last entry the errors were evaluated from well-defined imprecisions, and do not differ in their nature from the imprecisions in Part A. These imprecisions are combined in a sum of the squares addition with those of Part A. In a more restricted definition, we are referring to systematic errors as those which introduce a bias into the results but which are not well enough investigated to be evaluated by statistical techniques, or are not realized to be important. The limits of a systematic error are apt to be less well-defined than for a random error, although there must be some theoretical or experimental results upon which an estimate can be based.

We attributed an element of systematic error to the last entry in Part B, inasmuch as neither theory nor experiment for thermomolecular pressure effects is complete. Another important systematic error might be the effect of contaminating gases on the measured pressure. We believe our equipment and procedures were devised in such a way that we were able to prove that this error could not have been significant in our measurements.

The combination of the various random errors for 99 percent confidence limits requires that they be weighted differently according to the number of degrees of freedom. The random errors in Part A were applicable to the 5 final pressures of table 9. With 4 degrees of freedom, the standard deviations were multiplied by 4.6 as given in Student's Table [20], to compute the limits for 99 percent confidence. In Part B, the number of degrees of freedom is nine or more, so that a multiplier of 3.2 was used. The separate weighted standard deviations of Part A and Part B were combined by the sum of the squares, to give an estimated uncertainty of ±10 mPa at the 99 percent confidence limits. The total additional systematic errors are estimated to be relatively insignificant.

## Figures and Tables

**Table 1:** Manometer pressure at the diaphragm on the manometer side

Set	*PRT* Capsule (Ω)	*t*_cap_ °C	Thermocouples				P_Man_ (Pa)
			U (*μ*V)	M (*μ*V)	L (*μ*V)	R (*μ*V)	
							
1	27.582052	19.9389	−3	21	4	−2	610.84306
2	27.582119	19.9395	−6	23	3	−4	610.84504
3	27.582445	19.9428	−5	25	5	−3	610.84572
4	27.583308	19.9513	−6	24	4	−5	610.84403
5	27.583481	19.9530	−8	21	4	−6	610.84662

**Table 2:** Observations of manometer zero

Date	PRT Capsule (Ω)	*V_UC_* (*μ*V)	*V*_LCL_ (*μ*V)	*V*_LCR_ (*μ*V)	*C*_var_ (pF)	Calc *P* (Pa)	Corr (nm)	Corr C_Var_ (pF)
								
11/27								
11:21	27.583362	−16	−5	−21	0.02323	4.04 × 10^−3^	+ 30	0.02353
1:45	27.581889	−17	−6	−18	.02362	7.15 × 10^−3^	+ 54	.02416
2:55	27.581750	−3	3	−8	.02428	1.29 × 10^−3^	+ 10	.02438
4:15	27.581966	−9	0	−12	.02409	4.88 × 10^−3^	+ 37	.02446
5:17	27.581802	−14	−3	−16	.02391	6.83 × 10^−3^	+ 51	.02442
12/3								
10:10	27.593201	−17	−6	−24	.02356	2.24 × 10^−3^	+ 17	.02373
2:03	27.591870	−18	−6	−21	.02357	6.33 × 10^−3^	+ 48	.02405
12/4								
9:25	27.587772	−17	−5	−20	.02343	6.49 × 10^−3^	+ 49	.02392
12/5								
9:50	27.584449	−17	−5	−19	.02346	7.31 × 10^−3^	+ 55	.02401
12/6								
11:41	27.582260	−15	−5	−18	.02333	4.87 × 10^−3^	+ 37	.02370
							Ave 0.02404 Calib. Corr + 0.00031	
								Net 0.02435	

The estimate of the standard deviation of the mean is *S =* 0.00012 pF.

**Table 3:** Pressure effect from imperfect gaging joints

Set	*dp/dt* (*μm* Hg/min)			δP_Wring_
	LCL	LCR	UC	(Pa)
				
Zero Level	15	39	5	
1, 2 and 3	16	43	23	0.00373
4 and 5	16	40	22	0.00357

**Table 4:** Measurement scheme to determine manometer capacitance characteristics

Figure	Switch Pos.	Tap	Center Tap	*C*_Var_	Capacitance Equation
					
3a	I	*R*_2_	Gnd	Out	*C*_I_ *= C*_II_
3b	II	*R*_2_	Gnd	Out	*C_II_ = C_I_*
3c	I	*R*_2_	UnGnd	0.04313 pF Corr. 0.00055 pF	*C*_T_ + *C*_I_ = *C*_II_ + *C*_Var_*C*_T_ = 0.04368 pF
3d	III	*R*_2_	UnGnd	Out	*C*_T_ + *C*_UC_^=^ *C*_II_ *C*_UC_ = 8.16092 pF
3e	IV	*R*_1_	UnGnd	0.02373 pF Corr. + 0.00006 pF	*C*_T_ + Σ*C*_LC_ = 3/4 (*C*_UC_ + *C*_Var_) Σ*C*_LC_ = 6.13853 pF − 0.04368 pF
				.02379 pF	Σ*C*_LC_ = 6.09485 pF
3f	VI	*R*_2_	UnGnd	0.01215 pF Corr. + .00012 pF	*C*_T_ + *C*_LCR_ + *C*_Var_ = *C*_LCL_
				0.01227 pF	*C*_LCR_= *C*_LCL_ −0.05595 pF *C*_LCL_ = 3.07540 pF *C*_LCR_ *=* 3.01945 pF

**Table 5:** Characteristics of manometer cells

	Cap.^a^	Eff. Diam.	S*_0_*
			
Cell	(pF)	(cm)	(*μ*m)
UC	8.16092	3.07838	807.517
LCL	3.07540	1.88488	803.364
LCR	3.01945	1.88509	818.432

a The number of digits is consistent with the imprecision. The total uncertainty is nearly 1 part in 10^4^, but the net changes are known within 3 ppm of the total figure.

**Table 6:** Pressure increment determined by capacitor setting

Set	*C*_Var_ (pF)	Δ*C* (pF)	*d* (*μ*m)	δ*P*_CVar_ (Pa)
				
1	0.02644	0.044703	5.9911	0.79532
2 and 3	.02531	.043573	5.8380	.77507
4 and 5	.025306	.043569	5.8376	.77502

**Table 7:** Thermomolecular pressure correction

Set	*t*_ave_ (°C)	Δ*p* (Weber) (Pa)	0.76 Δ*p* (Pa)
			
1	25.05	0.02122	0.01613
2	25.13	.02155	.01638
3	25.13	.02155	.01638
4	25.03	.02105	.01600
5	24.82	.02021	.01536

**Table 8:** Measured pressure differences at the diaphragm

Set	Ave Defl (div)	δ*P*_DIA_ (Pa)	*S* (div)	*S* (Pa)	*S_m_* (div)	*S_m_* (Pa)
						
1	−3.82	−0.0205	1.65	0.0089	0.52	0.0028
2	1.38	.0074	1.31	.0070	.43	.0023
3	−0.12	−.0006	1.04	.0056	.35	.0019
4	.82	.0044	1.79	.0091	.52	.0028
5	.89	.0048	1.39	.0075	.28	.0015

**Table 9:** Total values of the vapor pressure of water at its triple point

Set	*P*_Man_ (Pa)	δ*P*_Wring_ (Pa)	δ*P_C_*_Var_ (Pa)	δ*P*_Thermp_ (Pa)	δ*P*_DIA_ (Pa)	δ*P*_VapHd_ (Pa)	*P*_tp_ (Pa)
							
1	610.84306	0.00373	0.79532	0.01613	−0.0205	0.01424	611.6520
2	610.84504	.00373	.77507	.01638	.0074	.01424	611.6619
3	610.84572	.00373	.77507	.01638	−.0006	.01424	611.6545
4	610.84403	.00357	.77502	.01600	.0044	.01424	611.6573
5	610.84662	.00357	.77502	.01536	.0048	.01424	611.6596

The average of the 5 sets is 611.6571 Pa (*S* = 3.94 mPa). The estimate of the standard deviation of the mean is *S_m_* = 1.76 mPa.

**Table 10:** Isotopic composition of water in the vapor pressure cell

	Sample No.	Description	D/H%	D(ppm)	0_18_/0_16_ %	0^18^ (ppm)
						
1st cell	1	raw (tap) water	−4.0	149	−0.58	1983
	2	Beginning of cell filling	−4.9	148	−0.71	1980
	3	End of cell filling	−5.6	147	−0.88	1977
2nd cell	4	Beginning of cell filling	−4.6	148	−0.60	1983
	5	raw (tap) water	−5.5	147	−0.77	1980
	6	End of cell filling	−5.1	148	−0.64	1982
	7	Distilled from apparatus 2 weeks after experiment	−6.9	145		
						
Average				147		1982

**Table 11:** Sources of error

(A) Random errors Observable in the Final Values	(Pa)	
Estimate of the standard deviation in the pressure as a result of imprecision in thermocouple e.m.f.'s	*S =* 0.0020	
Estimate of the standard deviation in assignment of the diaphragm zero (1/2 div.)	*S =* 0.0027	
Estimate of the standard deviation of the pressure head because of uncertainty in the location of the triple phase line (within 2 cm at 99% confidence)	*S =* 0.0003	
Estimate of the standard deviation of the pressure determination (the weighted average of the standard deviations of the diaphragm readings for the 5 sets)	*S =* 0.0019	
The square root of the sum of the squares of this group is 0.0039 Pa and agrees with the imprecision of the results in table 9 expressed as the estimate of a standard deviation of a single measurement. The estimate of the standard deviation of the mean for 5 sets is 0.0017 Pa.		
(B) Random Errors and Systematic Errors Not Observed as Imprecision in the Results		
Estimate of the standard deviation of the mean of the manometer zero (based on 10 determinations)	*S_m_ =* 0.0016	
Estimate of the standard deviation of the mean of the gage block calibrations (based on 20 determinations)	*S_m_ =* 0.0003	
Estimate of the standard deviation of the mean of calculating the effect of imperfect gage block joints (based on the standard deviation of the calibration)	*S_m_ =* 0.0005	
Estimate of the error from the variable capacitor calibration (based on comparisons with a capacitor calibrated with 10 ppm total uncertainty)	*S* = 0.0002	
Estimate of the error in calculating the pressure from the variable capacitor readings (based on the measurement uncertainties of the apparatus dimensions)	*S* = 0.0005	
Estimate of the error in the calculation of the thermomolecular pressure effect (the random error based on the standard deviation of the calibration)	Random *S_m_* = 0.0003 Systematic 0.0003	
The sum of the random errors in Part B, combined by the square root of the sum of the squares, is 0.0018 Pa. There is also a systematic error of 0.0003 Pa.		

## Footnotes

1 Also it is the terminus of the ice I-liquid curve, but this is of no interest for vapor pressure measurements.

2 Figures in brackets indicate the literature reference at the end of this paper.

3 Based on the equivalence of 760 mm Hg = 101325 Pa.

4 Certain commercial materials and instruments are identified in this paper in order to adequately specify the apparatus and experimental procedure. Such identification does not imply recommendation or endorsement by the National Bureau of Standards.

5 The calculated variation of the temperature of the ice mantle over its length because of the fluid head was less than 1 *μ*K.

## Appendix I Fortran IV program for calculating t_68_ from PRT measurements

100 * FOPTRAN IV PROGRAM NAMED BRIDGE FOR CALCULATION OF
110 * IPTS-68 VALUES FROM PRT RESISTANCE MEASUREMENTS.
120 DOUBLE PRECISION RT (5), R (5), W, A (10), B (10), T (5), T1, Z
130 DIMENSION N (5)
140 CALL DEFINE (1, 7HTCAL 74,)
150 CALL DEFINE (2, 4HPRT,)
160 READ (1, 110) (a (j), b (j), j = 1, 10)
170 110 FORMAT (D15.8, 2X, D15.8)
180 120 READ (2,130) (S1, S2, (N (J), RT (J), R (J), J = 1,4))
190 130 FORMAT (2X, F6. 2, X, F6. 3, (4 (/2X, 14, D15. 8, D15. 8)))
200 WRITE (9, 130) (S1, S2, (N (J), RT (J), R (J), J = (1, 4))
210 WRITE (9, 150) S1, S2
220 150 FORMAT (/14X, ‘STATE’ F6.2, 2X, F6.3,/)
230 DO 240 J = 1, 4
240 W = RT (J) / (R(J) - 0.996D-03)
250 I = N (J) - 700
260 T (J) = (-A (I) + DSORT (A (I) ** 2–4.* (1. -W) * B (I))) /(2. * B (I))
270 Z = 0.45D-03* T (J) * (T (J)/ 100.-1.) * (T (J) / 419.58-1.) * (T (J) / 630. 74-1.)
280 T (J) = T (J) + Z
290 WRITE (9, 230) N (J), T (J)
300 230 FORMAT (‘THERM. NO. ‘I 3,3X,’ T (INT. 1968) = ’ D17. 10)
310 240 CONTINUE
320 T1 = (T (2) + T (3) + T (4)) / 3
330 WRITE (9, 270) T1
340 270 FORMAT (‘AVE. T (INT. 1968) = ’ D17. 10, /, /)
350 GO TO 120
360 END
TCAL74

**Table:**

0.39849575D-02	−0.58762415D-06
0.39850801D-02	−0.58772119D-06
0.39848143D-02	−0.58762185D-06
0.39849917D-02	−0.58769654D-06
0.39849663D-02	−0.58756733D-06
0.39847884D-02	−0.58757724D-06
0.39848898D-02	−0.58760947D-06
0.39850236D-02	−0.58778636D-06
0.39838174D-02	−0.58757363D-06
0.39855387D-02	−0.58755669D-06

## Appendix II Basic Program for Calculating Pressure From Manometer Readings

100 REM BASIC PROGRAM NAMED “PRSURE”
110 REM TO CALCULATE EQ. 27 FOR MANOMETER.
120 REM UNITS ARE SI, RESULTS IN PASCALS.
130 READ R0,G1,M,H0
140 DATA 1.354582E 04,9.801022,0.40026E-2,5.176
150 DATA 486, 1013,405,486
160 FOR I =1T04
170 READ X(I)
180 NEXT I
190 READ S1,S2,T0,H2
200 FOR I=1 TO 4
210 READ E(I)
220 NEXT I
230 FOR I=1TO4
240 LET T(I)=E(I)/X(I)
250 LET T(I) = T(I)+T0
260 NEXT I
270 T1 = (T(3) + T(4))/2
280 P=0.8079*(T1-T(1))
290 P=P+0.0147*(T(1)-20)
300 P=P+R0*G1*H2*(1-0.17255E-3*(T(2)-20))
310 LET P=P+R0*G1*H2*(2.27E-6*H2-2.5E-7-4.07E-3*M*H0)
315 P=P+.171
320 PRINT S1; S2;P
330 GO TO 190
340 DATA 1.34, 1.1, 19.9389,4.6000292E-3
350 DATA -3,2 1,4,-2
360 DATA 1.34, 1.2, 19.9395,4.6000292E-3
370 DATA -6,23,3,-4
380 DATA 1.34, 1.3, 19.9428,4.6000292E-3
390 DATA -5,25, 5,-3
400 DATA 1.34, 1.4, 19.9513,4.6000292E-3
410 DATA -6,24,4,-5
420 DATA 1.34,1.5,19.9530,4.6000292E-3
430 DATA -8,2 1,4,-6
540 END

**Table:**

1.34	1.1	610.84306171
1.34	1.2	610.84503579
1.34	1.3	610.84572239
1.34	1.4	610.84402799
1.34	1.5	610.84661895

## Appendix III Basic Program For Calculating Pressure Effects at Zero Level

100 REM BASIC PROGRAM NAMED “ZPRES”
110 REM TO CALCULATE EQ. 27 FOR MANOMETER ZERO.
120 REM UNITS ARE SI, RESULTS IN PASCALS.
130 READ R0, G1, M, H0
140 DATA 1.354584E 04,9.801022,0.40026E-2,5.176
150 DATA 486,1013,405,485
160 FOR I =1TO4
170 READ X(I)
180 NEXT I
190 U0 = 20
200 READ S1, S2, T0
210 FOR I=1 TO 4
220 READ E(I)
230 NEXT I
240 FOR I= 1TO4
250 LET T(I) = E(I)/X(I)
260 LET T(I) = T(I)+T0
270 NEXT I
280 T1 = (T(3)+T(4))/2
290 P= 0.8079* (T1-T(1))
300 P=P+0.0147*(T(1) - T1)
310 PRINT S1; S2;P
320 GO TO 200
330 DATA 1048,1.0,19.9567
340 DATA - 16, 5,-5,-21
350 DATA 1048,1.1,19.8094
360 DATA - 17,7,-6,- 18
370 DATA 1048,1.2,19.7855
380 DATA -3, 19, 3,-8
390 DATA 1048,1.3,19.8169
400 DATA -9, 11, 0,-12
410 DATA 1048,1.4,19.8007
420 DATA - 14, 8,-3,-16
430 DATA 1048,1.5,20.0502
440 DATA -17, 7,-6,-24
450 DATA 1048,1.6,20.0369
460 DATA -18,6,-6,-21
470 DATA 1048,1.7,19.9959
480 DATA - 17,7, - 5, -20
490 DATA 1048,1.8,19.9627
500 DATA - 17,9,-5,-19
510 DATA 1048,1.9,19.9408
520 DATA -15,7,-5,-18
530 END

**Table:**

1048	1	.404552E-02
1048	1.1	.715303E-02
1048	1.2	.129202E-02
1048	1.3	.487762E-02
1048	1.4	.682625E-02
1048	1.5	.224516E-02
1048	1.6	.633001E-02
1048	1.7	.649 643E-02
1048	1.8	.731340E-02
1048	1.9	.48 68 54E-02

## Appendix IV Basic Program For Calculating Thermomolecular Pressure

100 REM BASIC PROGRAM “THERMP”, DERIVED FROM WEBEP-SCHMIDT EQ. FOR
110 REM CALCULATING THE THERMOMOLECULAR PRESSURE DEVELOPED BY
120 REM HELIUM IN A TUBE OF RADIUS R RUNNING BETWEEN TEMPERATURES OF
130 REM T1 TO T2. UNITS CGS, PRESSURE IN DYNES/CM† 2.
140 READ L0,T0,K1,K2,N0,N
150 READ P,M,R0
160 DATA 17.85, 273. 15, 1.36, 1.68, 1.86S85E-04, 0. 1866
170 DATA .1492, 1.25, 1.7846E-10
180 P=6116.4
190 PRINT “P = ” P
200 READ T2,T1
210 T1=T1+273.15
220 T2 = T2+ 273.15
230 PRINT “T1 = ” T1,“ T2 = ” T2
240 LET S=(T1/T2)**(N+1)
250 LET S0=(T1/T0)**(N+1)
260 LET L1 = L0*S0
270 LET E=4*K2*L1/R
280 LET F=M-1 / (2*(N+ 1))
290 LET D= 3* F*K1/(2*3.14159 *K2)
300 LET G = 0.5*((E/P)†2)*(1+S†2)*)(1-D)
310 LET H=2*E*(1 + S†2/(S+1))/(3*P)
320 LET Q= 1 -H+ G
330 LET U=(T1**(2+2*N)-T2** (2+2*N))/(TO**(2+2*N))
340 LET W=6*K1*(N0†2)*U*Q
350 LET V=(1+N)*R0*R†2
360 LET X =W/V
370 PPINT “P1 † 2 -P2† 2 = ” X
380 LET P3=X/(2*P)
390 LET Y=P/(P+P3/2)
400 LET P3= P3*Y
410 PRINT “DELTA P =” P3
420 PRINT
430 GO TO 200
440 DATA 19.94, 25.05, 19.94, 25.13, 19.96, 25.03
450 DATA 19.95, 24.82
460 STOP
470 END

**Table:**

?XBASIC THEFMP DB		
RUN		
P = 6116.4		
T1 = 298.2	T2 =	29 3.09
P1† 2 -P2†2 =	2595.48711	
DELTA P =	.21217073	
T1 = 298.28	T2 =	29 3.09
P1† 2 -P2†2 =	2636.439 1679	
DELTA P =	.21551834	
T1 = 298.18	T2 =	293. 11
P1† 2 -P2†2 =	2575.2255458	
DELTA P =	.21051446	
T1 = 297.97	T2=	293.1
P1† 2 -P2†2 =	2472.7760644	
DELTA P =	.20213976	
